# How Environmental Leadership Boosts Employees’ Green Innovation Behavior? A Moderated Mediation Model

**DOI:** 10.3389/fpsyg.2021.689671

**Published:** 2022-01-27

**Authors:** Binfeng Xu, Xiaopei Gao, Wenjing Cai, Lin Jiang

**Affiliations:** ^1^School of Public Affairs, University of Science and Technology of China, Hefei, China; ^2^Intellectual Property Research Institute, Hefei, China; ^3^Department of Management and Organization, Vrije Universiteit Amsterdam, Amsterdam, Netherlands

**Keywords:** environmental leadership, green organizational identity, green organizational climate, green innovation behavior, moderated mediation

## Abstract

We examined employees’ green organizational identity as a mediator and green organizational climate as a moderator in the relationship between environmental leadership and follower green innovation behavior. Through collecting data (*N* = 313) from public organizations in China at different times, we found that environmental leadership is positively related to employees’ green innovation behavior through increasing their green organizational identity. Meanwhile, the mediating relationship is conditional on the moderator of green organizational climate. The current study aims to clarify the mechanism and boundary condition in the relationship between environmental leadership and employees’ green innovation behaviors.

## Introduction

With the developments of society and economy, practitioners are calling for the awareness and implementation of environmental protection (Dangelico and Pujari, 2010; Guo et al., 2018; Cai et al., 2020). Indeed, improvements in most organizations are increasingly requiring their employees to be environmental in the workplace, such as displaying recycle behaviors, and saving energy in the office (Norton et al., 2017; Zhang et al., 2018; Kim et al., 2019). This line of research suggests that employees’ adoption of new practices and behaviors relating to protect environment is essential to the realization of organizational environmental strategy (Boiral, 2009). In this regard, researchers in the environmental management areas adopt a “behavioral perspective” among workforce including leaders and followers (Norton et al., 2015; Kim et al., 2019). That is, sustainability practices can develop an environmental atmosphere to further reinforce a green-oriented sense among individuals (e.g., employees, leaders, and customers) (Sowamber et al., 2017; Dewnarain et al., 2019; Ramkissoon et al., 2020). For example, scholars have highlighted the role of leaders’ green and environmental supervisory behaviors and leadership styles in boosting followers’ green and environmental outputs.

**FIGURE 1:**
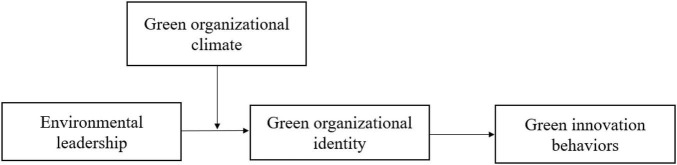
The hypothesized model.

However, despite accumulating evidence of the positive relationship between such leadership styles as transformational leadership and ethical leadership and employees’ pro-environmental behaviors, there is limited empirical research regarding to the potential impact of environmental leadership on employees’ green innovation behaviors in the workplace. Second, to comprehensively understand the linkage between environmental leadership and employee green innovation behavior, we draw on social identity theory (Stets and Burke, 2000)—suggesting that individuals’ self-identity and self-concept can be adapted by organizations toward corresponding individual behavioral and attitude outcomes (Hogg et al., 1995)—to propose employees’ green organizational identity as a mediator to link the environmental leadership and employees’ green innovation behaviors. Moreover, considering the fact that the effectiveness of leadership styles depends on come contextual factors, we follow previous studies that claim the desirable organizational climate can strengthen the influences of leadership on employee’s outcomes (Norton et al., 2012; Zientara and Zamojska, 2018) to expect that green organizational climate can positively moderate the relationship between environmental leadership and employee green innovation behavior via green organizational identity. Taken together, we in the current study aim to answer the question of *how and when does environmental leadership trigger green innovation behaviors among followers*. Figure 1 shows our hypothesized model.

This current study aims to make several contributions to the existing literature. First, we are among the first attempts to link environmental leadership and followers’ corresponding behavioral outcomes (i.e., green innovation behaviors). Second, we unfold the mechanisms through which environmental leadership exerts an effect on followers’ green innovation behaviors. By applying social identity theory, we contribute to the perspective of identity as a potential mediator in transmitting the effect of leadership and employees’ behaviors in a green way. Finally, through exploring the boundary condition (i.e., green organizational climate) on the relationship between environmental leadership and employees’ green innovation behavior, we enrich the current understanding of the effectiveness of environmental leadership in the workplace. In this vein, we provide empirical evidence to the theoretical arguments on the function of personal and situational attributes during interactions in the leadership literature.

## Theoretical Background and Hypotheses Development

### Environmental Leadership and Green Organizational Identity

Drawing on the perspective of natural resource-based view theory, we expect a positive influence of environmental leadership on follower’s identification. Specifically, contrary to traditional leadership styles, the approach of environmental leadership highlights the importance of environmental issues by enacting resource-valued practices (Tãpuricã and Ispasoiu, 2013). That is, environmental leaders aim to resolve conflicts between the organizations and the environment by identifying environmental problems and formulating environmental strategies. In addition, they emphasize on communicating with other organizations, and interpreting and solving environmental problems (Wang et al., 2020).

According to the social identity theory, we propose the benefits of environmental leadership on employees’ organizational identity based on the following reasons. First, organizational identity is formed in the leader’s understanding and belief of problems; therefore, he/she guides and promotes corresponding organizational behavior (Lede et al., 2019). The core of environmental leadership lies in the communication between leaders and followers, and even the exchange of rights beyond their respective power boundaries. The influence of environmental leadership on employees, thus, is expressed in some form within the organization (Fatoki, 2019); as a result, the consensus of organizational identity among employees would be developed. Second, since leader acts as a key role in the organization, when they enact environmental behaviors, environmental problems would be successfully solved through the communication, understanding and cooperation between leaders and organization members on environmental issues (Robertson and Barling, 2013; Chen et al., 2014). In this situation, employees will be highly attached to these environmental values through the organizations; therefore, the formation of green organizational identity will be realized among employees. Third, previous studies have shown that environmental leadership can be recognized as a background symbol of green organization identity, which affects the cognition and thinking of followers on environmental issues (Scott, 2007). Specifically, environmental leaders not only affect followers’ common values, commitments and aspirations to deal with environmental problems, but also increase their passion for environmental protection. As a result, these followers’ green identification with their organizations is likely to be enhanced. Taken together, we propose the following hypothesis:

*H1. Environmental leadership is positively related to green organizational identity.*

### Green Organizational Identity and Green Innovation Behaviors

Organizational identity represents a motivating factor that affects the behavior of employees, which is theoretically a way to describe organizational characteristics or a real phenomenon established by individuals or teams through symbols and languages. According to the organization identity theory, green organization identity exerts a positive impact on the environmental behavior of organizations (Chang et al., 2019). When employees have a positive identification with the way their organizations deal with environmental issues, they tend to build an emotional connection which encourages the organization to view environmental protection as an opportunity rather than a threat toward improving organizational competitive advantage (Ernst and Jensen, 2019). Furthermore, under the encouragement of common green organization identification, employees explore the connection between green innovation technology and customers’ environmental needs, and solve environmental problems through green product and/or process innovation.

Green organization identification can stimulate employees to comprehensively improve the quality of green products and promote the efficiency of coordinated environmental development. Employees’ positive cognition on and participation in environmental problems through integration and utilization of new knowledge and new ideas can be significantly improved (Song and Yu, 2018). Therefore, we propose the following hypothesis:

*H2. Green organizational identity is positively related to green innovation behaviors.*

### Mediator of Green Organizational Identity

Although it is widely acknowledged that the possibility and enthusiasm of green innovation performance depends on whether the environmental leader involves employees in the decision-making of the organizational environmental behavior, we further propose that green organization identification plays a key role in these processes, because the implementation of the specific content of green innovation depends on employees. Specifically, employees’ environmental-oriented organizational identity can influence their understanding of their organizational environmental strategic issues (Chen, 2011); therefore, they are more likely to engage in green activities in an innovative way (Song and Yu, 2018). In addition, leadership plays a key role in shaping followers’ cognitions, such as, the thinking mode of environmental management in organizations (Carmeli et al., 2017). Thus, they establish a common cognition of environmental management and green innovation, and thus improves the performance of green innovation. Taken together, we propose the following hypothesis:

*H3. Organizational identity mediates the relationship between environmental leadership and green innovation behaviors.*

### Moderator of Green Organizational Climate

Due to the fact that the effectiveness of leadership is contingent on the organizational environment (Kerr and Jermier, 1978), we also propose that green climate acts as a moderator in the relationship between environmental leadership and employees’ organizational identity. Theoretically, organizational environment is a shared personal value that involves the construction of the meaning of the work environment (James et al., 2008), where employees are expected to act in accordance with the influence of specific social constructions imposed in the working environment) (Bellou and Andronikidis, 2009). Relating to the green literature, the green organizational climate is considered a subtype of the organizational climate, in which the corporation focuses on the implementation of environmental policies and measures and the employees may form a shared value regarding corporate greening.

In the situation where employees perceive their organizational environment as being green, their attitudes would be influenced by the climate of being green (Norton et al., 2015). Such green climate provides a strategic focus for leaders’ behaviors, and enables leaders to be more effective at directing employee cognitions toward achieving environmental outputs (Zientara and Zamojska, 2018; Song et al., 2020). That is, employees tend to develop their identification with their organizations in an environmental manner, which facilities their green organizational identity. In contrast, when the green organizational climate is low, employees may feel that being environmental is not appreciated and valued in the organization. As a result, they may not receive the environmental information from their leaders, and then fail to build their green organizational identity. Therefore, we propose the following hypothesis:

*H4. Green organizational climate positively moderates the relationship between environmental leadership and green organizational identity, such that the relationship is stronger when green organizational climate is high than when it is low.*

### Moderated Mediation Model

Furthermore, given the theoretical arguments above, we propose a moderated mediation model; that is, the relationship between environmental leadership and employee green innovation behaviors through organizational identity can be moderated by green organizational climate. Since green initiatives respond to demands for more ethical corporations, when employees are working in an environment where organizations support the environmental endeavors, they are more likely to receive more information about being environmental. Thus, they would trust their leaders’ environmental behaviors as they know these behaviors are consistent with the organizational values. In this vein, employees develop their organizational identity in an environmental way and then enact green innovation behavior in the workplace. Taken together, we propose the following hypothesis:

*H5. Green organizational climate positively moderates the relationship between environmental leadership and employee green innovation behaviors through green organizational identity, such that the relationship is stronger when green organizational climate is high than when it is low.*

## Materials and Methods

### Samples and Procedures

We used a survey questionnaire research design in the study. We collected the data from governments in the mainland China at two different times. Specifically, before submitting questionnaires, we first received the willingness of participating in our research from 10 governmental sectors; then, we submitted our questionnaires to employees working in these governments who would like to fill in the questionnaires voluntarily at Time 1. They completed the questionnaires during their working hours about environmental leadership, green organizational climate, green organizational identity; afterward, they sent their results to the authors directly. We submitted 419 questionnaires and received 357 questionnaires. One month later, at Time 2, we submitted another set of questionnaires to employees to rate their own green innovation behaviors. After deleting the questionnaires with incomplete information, the final sample consisted of 298 employees. Among them, 64.94% were female; 74.16% had obtained at least a bachelor’s degree; and 63.91% had an organizational tenure of more than 5 years. Their average age was 30.09 (SD = 7.25) years old.

### Measures

All the scales used in the current study are from previous studies and have been validated in related research. Since the original scales are English scales, we used a back-translation method to provide a Chinese version (Brislin, 1980). Seven-point Likert scales (from 1 = strongly disagree, to 5 = strongly agree) were used.

#### Environmental Leadership

We used a 4-item scale from Tãpuricã and Ispasoiu (2013) to measure environmental leadership. A sample item is “My leader encourages environmental sustainability, creates and maintains green values as a shared vision of the organization.” The Cronbach’s α is 0.90.

#### Green Organizational Identity

We used a scale with 6 items from Chang and Chen (2013) to assess green employees’ organizational identity. A sample item is “I feel that the company have formulated well-defined environmental goals and missions.” The Cronbach’s α is 0.82.

#### Green Organizational Climate

We used a 25 item scale from Norton et al. (2017) to assess employees’ green innovation behaviors. An example item was “My organization is concerned with becoming more environmentally friendly.” The Cronbach’s α is 0.83.

#### Green Innovation Behaviors

We used a 8-item scale from Chang and Chen (2013) to assess employees’ green innovation behaviors. A sample items is “I choose the materials of the product that produce the least amount of pollution for conducting product development or design.” The Cronbach’s α is 0.84.

#### Control Variables

Previous studies have indicated that demographic variables could exert an influence on individual green-related behaviors (Abrahamse and Steg, 2009; Dumont et al., 2017). Thus, we controlled the employees’ gender (1 = male; 2 = female), age (in years), educational level (1 = associate degree and below; 2 = bachelor’s degree; 3 = master and above), and work tenure (in years) in the current study.

## Results

### Validity Analyses

We first conducted a confirmatory factor analysis to validate the hypothesized model. Table 1 shows the results. Specifically, our hypothesized model fits the data better (χ^2^ = 256.81, *df* = 124.61; CFI = 0.98; TLI = 0.98; RMSEA = 0.02) than other alternative models (Hu and Bentler, 1999).

**TABLE 1:** Measurement model fit results.

Models	χ^2^	*df*	CFI	TLI	RMSEA
Hypothesized 4-factor model	256.81	124.61	0.98	0.98	0.02
3-factor model (Green organizational climate and green organizational identity combined)	947.62	132.94	0.80	0.80	0.08
2-factor model (Environmental leadership, green organizational climate and green organizational identity combined)	1362.83	139.35	0.76	0.77	1.11
1 factor-model (All variables combined)	2937.35	144.03	0.62	0.62	1.39
Hypothesized 4-factor model	256.81	124.61	0.98	0.98	0.02
3-factor model (Green organizational climate and green organizational identity combined)	947.62	132.94	0.80	0.80	0.08

Given the one-source data collection (i.e., all the data were collected from employees), we applied two methods to identify the potential for common method bias (CMB). First, according to the explanatory factor analysis (Harman, 1976), the results showed that one factor accounted for 34.66%, which is below the accepted threshold of 40%. Second, we conducted the test of the one-factor measurement model (Podsakoff and Dennis, 1986), which generated a poor fit to the data. Thus, CMB is not a serious problem in the current study.

Table 2 shows means, standard deviations, and inter-correlations of all the variables.

**TABLE 2:** Means, standard deviations, and correlations of studied variables.

Variables	Means	SD	1	2	3	4	5	6	7
1. Green innovation behavior	3.48	0.77							
2. Environmental leadership	3.97	0.75	0.34**						
3. Green organizational climate	4.01	0.80	0.27**	0.29**					
4. Green organizational identity	4.33	0.89	0.40**	0.36**	0.38**				
5. Age	30.09	7.25	0.08	0.00	0.04	0.03			
6. Gender	1.78	0.61	0.01	–0.01	0.00	0.04	0.01		
7. Educational level	2.31	0.97	0.05	0.02	0.04	0.00	0.10	0.06	
8. Work tenure	8.33	8.89	0.13	0.07	0.00	0.01	0.25**	0.02	0.00

N = 298. SD, standard deviation. Gender: 1 = male; 2 = female. Educational level: 1 = junior college or below; 2 = bachelor; 3 = master and above. *p < 0.05, **p < 0.01.

### Hypotheses Test

Confirmatory factor analysis was used to test for model fit for both the measurement and structural models. The estimates for the direct and indirect effects were provided for the structural equation model (Table 3) to address hypotheses concerning the direct relationship between environmental leadership and green organizational identity (H1), the direct relationship between green organizational identity and green innovation behaviors (H2), and the mediated relationships between environmental leadership and green innovation behaviors through green organizational identity (H3). The model showed acceptable fit to the data (χ^2^ = 299.31; *df* = 125; RMSEA = 0.03; GFI = 0.97; TLI = 0.97; CFI = 0.98). Thus, H1 which hypothesized that environmental leadership is positively related to green organizational identity was supported (β = 0.29, *t* = 2.47; *p* < 0.001). This finding is consistent with previous studies that such contextual factors as green-oriented leadership and leaders’ behaviors can be beneficial to the development of flowers’ identity (Wu et al., 1292). This implies that when employees perceive that their supervisors are enacting environmental leadership approach, they are likely to strengthen their green identity with their organizations. H2 proposes the direct effect of green organizational identity on green innovation behaviors. This hypothesis is also supported (see Table 3). The findings indicate that employees’ green organizational identity is a significant determinant of their green innovation behaviors (β = 0.31, *t* = 2.33; *p* < 0.001). H3 assumes the mediation effect of green organizational identity on the relationship between environmental leadership and green innovation behaviors. Results in Table 3 indicate that H3 receives support; that is, the indirect effect of environmental leadership on green innovation behaviors via green organizational identity is significant (β = 0.35, *t* = 3.66; *p* < 0.001). This suggests green organizational identity partially mediates this relationship, which helps enrich the current knowledge on multiple explanatory mechanisms to explain the influence of environmental leadership on green innovation behaviors. The relationships established using structural equation modeling replicate closely in Table 4 by the results using regression analysis. This provides a triangulation of the findings. The results in Table 4 take into account the control variables, and the results indicate that both environmental leadership (β = 0.19, *p* < 0.01) and green organizational identity (β = 0.37, *p* < 0.001) are positively associated with green innovation behaviors. Overall, these findings suggest that environmental leadership has a strong association with green innovation behaviors via green organizational identity, supporting all the hypotheses suggesting the mediation effect. specifically, these findings suggest that managers who display environmental leadership approach can significantly stimulate followers’ green innovation behaviors by stimulating their green organizational identity.

**TABLE 3:** Results of mediated effects.

Hypotheses	Direct effects	Indirect effects	Support/no support
H1. Environmental leadership→ Green organizational identity	0.29		Supported
H2. Green organizational identity→ Green innovation behaviors	0.31		Supported
H3. Environmental leadership→ Green organizational identity Green innovation behaviors		0.35	Supported

*N = 298. χ^2^ = 299.31; df = 125; RMSEA = 0.03; GFI = 0.97; TLI = 0.97; CFI = 0.98.*

**TABLE 4:** Results of the moderated regression analyses.

	Outcome variable: Green organizational identity				Outcome variable: Green innovation behaviors				Outcome variable: Green organizational identity			
	Coefficients	*SE*	*t*		Coefficients	*SE*	*t*		Coefficients	*SE*	*t*	
*Control variables*												
Age	0.02	0.01	1.41		0.00	0.05	0.09		0.04	0.01	1.12	
Gender	0.00	0.01	1.07		0.00	0.04	0.05		0.00	0.01	–0.10	
Educational level	0.02	0.01	0.98		0.01	0.02	0.02		0.05	0.04	0.46	
Work tenure	0.01	0.01	0.65		0.04	0.02	0.01		–0.01	0.03	–0.15	
*Independent variable*												
Environmental leadership	0.34**	0.09	2.62		0.19**	0.05	2.46		0.13**	0.04	–0.07	
*Mediator*												
Green organizational identity					0.37***	0.08	4.21					
*Moderator*												
Green organizational climate									0.40***	0.07	6.23	
*Interactive effects*												
Environmental leadership × green organizational climate									0.21***	0.02	4.39	
Model summary	*R*	*R* ^2^	*MSE*	*F*	*R*	*R* ^2^	*MSE*	*F*	*R*	*R* ^2^	*MSE*	*F*
	0.35	0.09	0.47	3.17	0.29	0.19	0.66	5.32	0.61	0.33	0.40	10.55

N = 298. *p < 0.05; **p < 0.01; ***p < 0.001.

**FIGURE 2:**
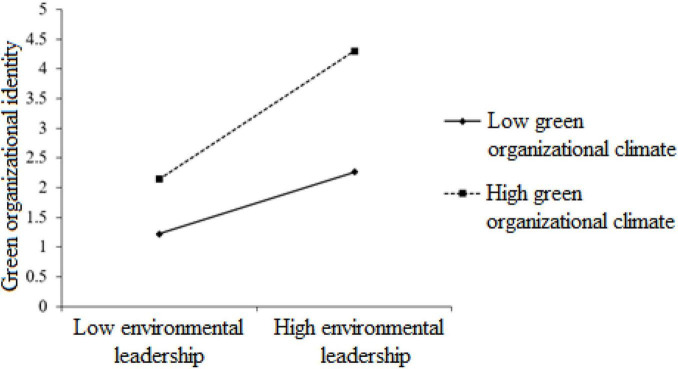
The moderating effect of green organizational climate on the relationship between environmental leadership and green organizational identity.

H4 testing for the moderating effects of green organizational climate on the relationship between environmental leadership and green organizational identity was undertaken using PROCESS in SPSS software from Hayes (2013). Model 7 was used to estimate the significance of the interaction terms. This was then followed by using ModProb (a freely available script developed by Preacher and Hayes, 2008). ModProb allows for probing interactions (Aiken et al., 1991) through generating data for graphical presentation. The results are presented in Table 4 and the graphical presentations are shown in Figure 2. The results indicate that green organizational climate is a moderator in the relationships between environmental leadership and green organizational identity (β = 0.19, *p* < 0.001). That is, green organizational climate can strengthen the influence of environmental leadership on employees’ green organizational identity such that when employees perceive a high level of green organizational climate, environmental leadership would exert a stronger effect on their green organizational identity. This is consistent with previous findings that positive working climate can reinforce the way of desirable leadership styles boosting followers’ corresponding outcomes in the workplace.

The results of the moderated mediation analyses testing H5 are presented in Table 5. To test the significance of conditional indirect effects of environmental leadership on employees’ green innovation behaviors through boosting their green organizational identity, we used boot estimates from the 10,000 bootstrap samples using PROCESS. The findings suggest that the conditional indirect relationship of environmental leadership with employees’ green innovation behaviors through green organizational identity was significant for the high level of green organizational climate (β = 0.09, *SE* = 0.05; 95% CI: 0.11, 0.15) compared with low level of green organizational climate (β = 0.03, *SE* = 0.01; 95% CI: 0.02, 0.09). Thus, H5 receives support.

**TABLE 5:** Results of the conditional indirect effects.

	Green organizational identity		
	Boot indirect. effect	Boot se	95% CI
High level of green organizational climate (1 + SD)	0.09	0.05	[0.11, 0.15]
Low level of green organizational climate (1−SD)	0.03	0.01	[0.02, 0.09]

## Discussion

### Theoretical Implications

In the current study, we examine the effects of environmental leadership on employees’ green innovation behavior through a mediator of green organizational identity, and conditioned by a moderator of green organizational climate. The results indicated that environmental leadership can boost employees’ green organizational identity and then green innovation behavior. In addition, when the green organizational climate is high, the mediated influences would be stronger. The study aims to make several contributions as followed.

First, the study is among the first to test the relationship between green-relevant leadership approaches and employees’ green-relevant outcomes (Fernández et al., 2006; Tãpuricã and Ispasoiu, 2013). We specify the benefits of environmental leadership for the increase of employees’ green innovation behaviors at the workplace. Although previous studies have indicated that when leaders display green-oriented supervisory behaviors or enact environmental-relevant leadership styles (Flannery and May, 1994; Chen et al., 2014; Wang et al., 2020), limited studies have empirically examined the positive effect of environmental leadership on followers’ green innovation. In this vein, our study enriches this line of literature by illustrating that employees’ green innovative outputs, such as behaviors, can be achieved by being supervised by an environmental leader (Fernández et al., 2006; Renwick et al., 2013; Norton et al., 2015).

Second, our findings of the mediator of green organizational identity extend the current understanding on how environmental leadership can boost employees’ green innovation behaviors (Chang and Chen, 2013). That is, the mechanism through which environmental leadership exerts an effect on employees’ innovation behaviors in a green way can be explained by the organizational identity theory. This specifically contributes to and significantly supports previous studies arguing for the possibilities of employees’ identity in transmitting the influences of leadership approaches to employees innovation in the green literature (Chen, 2011; Chang et al., 2019).

Finally, our exploration of the moderator of green organizational climate extends the leadership literature that the effects of leadership is contingent on contextual variables (Kerr and Jermier, 1978). The results show the boundary condition of environmental leadership influencing employees’ green innovation behavior through enhancing green organizational identity. This moderation effect provides novel and interesting insights into how a green organizational climate can strengthen the inducement mechanism of green behavior (Dumont et al., 2017; Zientara and Zamojska, 2018).

### Practical Implications

Based on these findings above, there are some practical implications. First, given the importance of being pro-environmental in the workplace, sustainability managerial practices should be enacted throughout the organizations. Meanwhile, it is highly suggested to build and develop employees’ and leaders’ awareness of engaging in green- and environmental-oriented practices during their working hours. For example, human resource department cannot only invest in providing “green” training to staff, but also focus on hiring and recruiting candidates with the mindset of being environmental.

Second, according to our results about the beneficial effect of green climate in the organizational on boosting the influences of leaders’ environmental behaviors toward employee’s green innovation behaviors, we recommend organizations to active green-relevant norms, which may stimulate both managers and followers to behave in an environmental manner toward the achievements of organizational sustainable goals.

### Limitations

Some limitations should be noted in the current study. First, our sample is only from China, thus, the generalizability of the findings should be extended through collecting data from other countries. Relatedly, the second limitation regards to the potential effects of variables about culture. Since we collected data in China where the collectivism is highlighted in most organizations, especially in the governmental sectors, future studies are encouraged to include some culture-relevant factors to further establish our research findings. Moreover, given the self-reported data, we encourage future studies to take objective assessments in order to establish our research findings. That is, obtaining third-party rating for some of the variables can be employed. For example, scholars could invite leaders to rate their own environmental leadership or their follower’s green innovation behaviors. Finally, although we used the time-lagged research design, we measured independent variable (i.e., environmental leadership, green organizational identity, and green organizational climate) at the same time (i.e., Time 1). Thus, we fail to address the problem of hypothesized causality. For example, when the organizational climate can be perceived as green-oriented, leaders who working in this situation would be likely to be influenced to develop their green leadership approach and green-related behaviors. Although we have conducted data analyses to justify that CMB is not a problem in our study, future research is still encouraged to use a longitudinal research design to acquire more reliable results. Relatedly, researchers can also conduct a three-wave of data collection, with environmental leadership, green organizational identity, and green innovation behaviors all measure at different points in time.

## Conclusion

Although previous studies have indicated the positive role of leaders’ environmental supervisory behaviors and leadership styles in facilitating employees’ environmental outcomes, limited empirical studies have been conducted to specifically test the potential relationship between environmental leadership and follower’s green innovation behaviors. In the current study, we aim to examine through what intervening mechanisms and under what boundary conditions environmental leadership can boost followers’ engagement in green innovation behaviors. Our findings reveal that when leaders enact environmental leadership approach, employees’ green innovation behavior would increase through enhancing their green organizational identity. Moreover, the findings show that green organizational climate can strengthen the mediating relationship. Taken together, this study illuminates why, how and when does environmental leadership can facilitate employees’ such desirable behaviors as green innovation behaviors.

## Data Availability Statement

The raw data supporting the conclusions of this article will be made available by the authors, without undue reservation.

## Author Contributions

BX and WC: conceptualization. WC and XG: methodology and validation. LJ: software. WC: formal analysis. BX: investigation. XG: resources. WC: data curation. BX, XG, and WC: writing—original draft preparation. All authors contributed to the article and approved the submitted version.

## Conflict of Interest

The authors declare that the research was conducted in the absence of any commercial or financial relationships that could be construed as a potential conflict of interest.

## Publisher’s Note

All claims expressed in this article are solely those of the authors and do not necessarily represent those of their affiliated organizations, or those of the publisher, the editors and the reviewers. Any product that may be evaluated in this article, or claim that may be made by its manufacturer, is not guaranteed or endorsed by the publisher.
